# Antimicrobial activities and in vitro properties of cold-adapted *Lactobacillus* strains isolated from the intestinal tract of cold water fishes of high latitude water areas in Xinjiang, China

**DOI:** 10.1186/s12866-019-1623-3

**Published:** 2019-11-07

**Authors:** Xiaojing Wei, Yan Zhang, Hong Zhou, Fengwei Tian, Yongqing Ni

**Affiliations:** 1 School of Food Science and Technology, Shihezi University, Fourth Nouth Ave., Shihezi, 832000 Xinjiang, People’s Republic of China; 2 School of Food Science and Technology, Jiangnan University, Wuxi, 214122 Jiangsu People’s Republic of China

**Keywords:** Cold-adapted, Rep-PCR, *Lactobacillus* spp., Probiotic characteristics, Antibiotics resistance, Cold-water fishes

## Abstract

**Background:**

There are still a large variety of microorganisms among aquatic animals, especially probiotic lactic acid bacteria in cold water fishes at high latitudes have not been fully developed. Hence, the present study aims to evaluate the probiotic potential of cold-adapted *Lactobacillus* strains isolated from the intestinal tract of cold water fishes (Xinjiang) and select candidates to be used as new food preservative agents and/or probiotic additives in feeding of aquaculture.

**Results:**

A total of 43 *Lactobacillus* spp. were isolated from 16 kinds of intestinal tract of cold-water fishes. They were characterized by phenotypic methods, identified using Rep-PCR and 16S rRNA gene sequencing as four species: *Lactobacillus sakei* (22 isolates), *Lactobacillus plantarum* (16 isolates), *Lactobacillus casei* (4 isolates) and *Lactobacillus paracasei* (1 isolate). The in vitro tests included survival in low pH and bile, antimicrobial activity (against *Escherichia coli*, *Salmonella enterica subsp. enterica serovar Typhimurium*, *Salmonella enterica subsp. enterica*, *Listeria monocytogenes*, and *Listeria innocua*), resistance to 15 antibiotics and hemolytic tests. Among all 43 lactobacilli isolates, the 22 isolates showed a wide range of antimicrobial activity against 6 different pathogenic strains. There were twenty isolates growing at optimal temperature ranging 16~20 °C, which were initially considered to be cold-adapted strains. Two (2) *Lb. sakei* strains and 2 *Lb. plantarum* strains demonstrated the highest survivability after 4 h of exposure at pH 2.0. Most of the tested strains cannot be cultured after exposed into 0.5% bile solution for 4 h, while 2 *Lb. plantarum* strains (E-HLM-3, CQ-CGC-2) and 1 *Lb. sakei* strain M-DGM-2 survived even at 2% bile concentration. In addition, the safety assessment showed that 22 strains without any detectable hemolytic activity and resistant to glycopeptides (vancomycin, teicoplanin), levofloxacin, aztreonam, amikacin and oxacillin, while all the studied lactobacilli showed sensitivity to or semi-tolerant to other antibiotics.

**Conclusions:**

Based on all the experiments, 3 strains, including E-HLM-3, CQ-CGC-2, and M-DGM-2 might be a candidate of choice for using in the food preservative agents and/or probiotic additives in feeding of aquaculture.

## Background

Lactic acid bacteria (LAB) are generally recognized as safe for use in foods. Again, most lactic acid bacteria also fulfill the QPS (qualified presumption of safety) requirements [1]. As the most distinguished probiotics within LAB, *Lactobacillus* has been extensively studied and widely used in food industry, animal husbandry, and health-care [2, 3]. In the nature, bacteria belonging to the genus *Lactobacillus* (lactobacilli) can be found in a variety of ecological niches such as animal/human gastrointestinal tract, insects, plants and raw milk. Thus, the genus *Lactobacillus* is a highly heterogeneous group of lactic acid bacteria (LAB) with important implications in food preservation, as starters for dairy products, fermented vegetables as well as animal husbandry. The ability to colonize a variety of habitats is a direct consequence of the wide metabolic versatility of this group of LAB.

Nowadays, the consumers increasingly demand for high-quality (nutritional aspects and food quality) and but minimally processed food [4]. The development of emerging lactobacilli preservatives as alternatives to chemical preservatives represents tremendous economic potential in the food industry [5]. Additionally, due to its large number and purported benefits for gut function and health, lactobacilli have also been used as probiotic additives in feeding, including animal husbandry and aquaculture. At present, more attention has been paid to lactobacilli isolated from mesophilic habitats, such as terrestrial animal gastro intestinal, dairy products, and fermented vegetables [6, 7]. In fact, there exists a kind of lactic acid bacteria growing at low temperature with a long history of use. These LAB can rapidly adapt to a temperature downshift and can continue to grow at a relatively higher rate at low cooling temperatures (4–10 °C), compared with LAB isolated from mesophilic habitats [8].

To date, most of recent reports concerning the lactic acid bacteria growing at low temperature are derived from processed meat and fish products during low temperature storage, and low temperature fermented vegetable [9]. LAB, include of *Lactobacillus* spp*.*, dominated vacuum-packaged cold-smoked fish products, for example. *Carnobacterium* spp. was quite common in chilled fresh and lightly preserved seafood [10]. Corresponding studies have shown that LAB present in food systems was able to exert antimicrobial activity and to inhibit the growth of food-borne spoilage organisms and pathogens [10, 11]. Hence cold-adapted LAB is more preferable candidates, and could meet the necessary requirements for biopreservation of fresh food products.

As is known to all, fish are poikilotherms, implying that their body temperature varies with ambient environments. Analogous to other vertebrate animals, LAB have been also found to be the micro-flora of fresh and sea water fish gut [12, 13]. Conceivably, fish-borne lactic acid bacteria are compatible to their host environment, and are able to strongly grow even at refrigerated temperatures. In comparison with fish living in tropic or temperate environment [14], fish inhabiting high altitude or latitude rivers and lakes on earth, called as cold-water fishes, often have a lower growth temperature ranging from 0 to 20 °C. Given the cold-water fishes coevolution with their corresponding gut bacterial community [14, 15], they are expected to provide a good survival circumstance for the cold-adapted bacteria, including psychrophilic and/or cold-adapted LAB. Therefore, LAB from fresh cold-water fishes may be of interest not only for food preservation, but also for aquaculture. However, such cold-adapted LAB have not been well studied and developed to date, especially those residing in the high latitude and/or altitude water areas.

The objective of this study was to identify to species level and evaluate the probiotic properties of cold-adapted *Lactobacillus* strains isolated from intestinal tract of the cold-water fishes, and select candidates to be used as new food preservative agents and/or probiotic additives in feeding of aquaculture. To the authors’ knowledge, this work is the first detailed report on the probiotic of the cultivable cold-adapted LAB within a given high latitude water areas in Xinjiang, China. We speculate that the cold-water fish intestine living in high-latitude lakes in Xinjiang is one of the good sources of cold-adapted lactic acid bacteria. The different habitats of the fish species might harbor distinctive assemblages of LAB. The goal, ultimately, is to identify which cold-adapted lactobacilli populations are have probiotic functions.

## Results

### Genotypic characterization

**Fig. 1:**
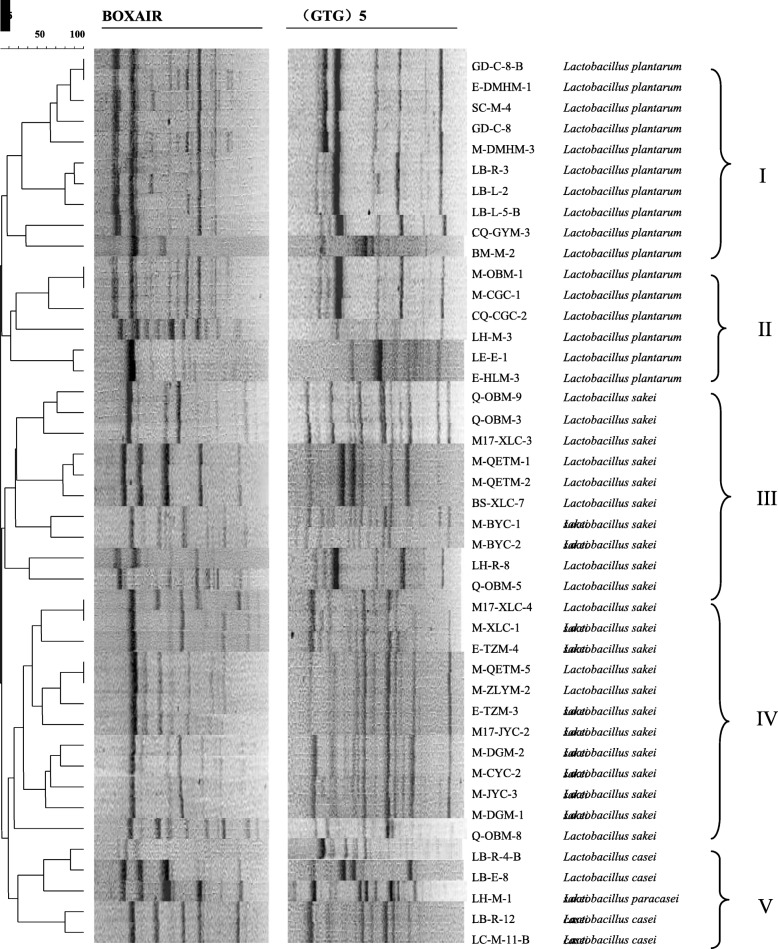
Dendrogram depicting of 43 *Lactobacillus* spp. strains according to the genetic profile obtained by Rep-PCR

A total of 135 pure cultures were isolated from the intestinal of cold-water fishes. Combining traditional strain identification methods with the 16S rRNA sequence analysis showed that 43 strains were *Lactobacillus* spp., including *Lactobacillus sakei* (22 isolates), *Lactobacillus plantarum* (16 isolates), *Lactobacillus casei* (4 isolates) and *Lactobacillus paracasei* (1 isolate). For further analysis, the Rep-PCR technique was used to evaluate the diversity of *Lactobacillus* spp. The results of agarose gel were analyzed by Gel Compar II 6.0 analysis software as shown in Fig. 1. The BOX-PCR and (GTG)_5_-PCR fingerprints clearly showed that the fingerprints of lactic acid bacteria had many different bands, which could reflect the differences in the genome level of different strains. The BOX-PCR fingerprints were mainly concentrated in the range of 400–5000 bp, including 3–11 bright bands and some weak bands; (GTG)_5_-PCR fingerprints had more specific bands in the range of 300–5000 bp, with 1–12 bands. Comparing the fingerprints of two different primers, it could be seen that the most of PCR bands were between 350 and 5000 bp.

As shown in the following figure, the 43 strains of *Lactobacillus* were clustered into 5 groups: *Lb. plantarum* was distributed in groups I and II, a total of 16 strains, accounting for 37% of *Lactobacillus* spp., *Lb. sakei* distributed in Groups III and IV, accounting for 51%. *Lb. casei* and *Lb. paracasei* were distributed in group V, accounting for 12% of *Lactobacillus* spp. Cluster I includes 10 *Lb. plantarum*, in which the strains CQ-GYM-3 and LB-R-3 (GTG)_5_ fingerprints were more distinctly from the other *Lb. plantarum*, another strain of *Lb. plantarum* BM-M-2 was also divided into this group, the fingerprint bands of the two primers were significantly different from other strains, indicating the unique genetic structure of the strain. Cluster II was also composed of 6 *Lb. plantarum* strains, but compared with the *Lb. plantarum* fingerprint pattern of group I, the difference between the bands was obvious, and LH-M-3 was the largest genetic difference of the group. Cluster III was composed of 10 strains of *Lb. sakei*, which could be further divided into four groups at 40% similarity level and the fingerprint pattern was similar between each group. Cluster IV consisted of 12 *Lb. sakei* and still could be further divided into four groups at 65% similarity levels. The fingerprint bands of each group were similar, but the strain Q-OBM-8 differs greatly from the other strains in its banding type alone. Cluster V was composed of 4 *Lb. casei* and 1 *Lb. paracasei*, among the 4 strains of *Lb. casei*, the LB-R-12 and LC-M-11-B strains were closed to each other, while the LB-R-4-B and LB-E-8 strains have obvious difference in spectrum, and the strain LH-M-1 has the largest genetic difference. Band-type statistics showed that 16 strains of *Lb. plantarum* had 6 types of bands, 22 strains of *Lb. sakei* had 8 types, 4 strains of *Lb. casei* had 3 types, and 1 strain of *Lb. paracasei* had only one bang type. In this study, 43 strains of *Lactobacillus* had rich genetic diversity, and these *Lactobacillus* species had obvious differences, with diversified intraspecies and extremely high genetic polymorphism. The Rep-PCR fingerprinting technology could well distinguish the *Lb. casei* and *Lb. paracasei.*

### Phenotypic characterization

**Fig. 2:**
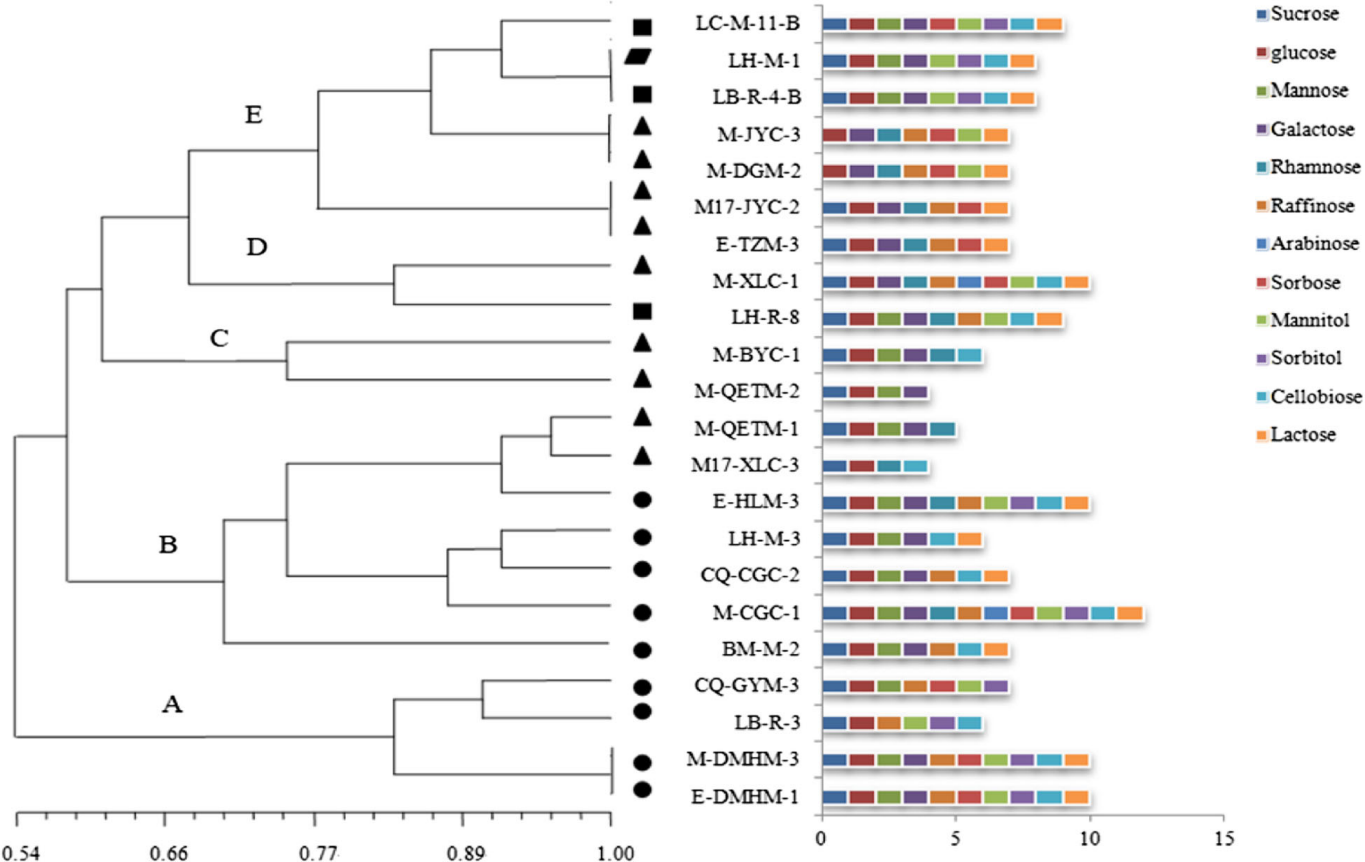
Dendrogram of 22 representative Lactobacillus strains isolated from the intestine of cold water fish based on sugar fermentation metabolism. *Lactobacillus casei*, *Lactobacillus paracasei*, *Lactobacillus sakei*, *Lactobacillus plantarum*

In a recent investigation, the interest for their identification was provoked by established antagonistic activity and their antibiotic susceptibility. The antibacterial ability of 43 lactobacilli was determined by Oxford Cup method, and 22 *Lactobacillus* strains with good antibacterial ability were screened out. All strains were screened according to their colony morphology (size, color, shape), gram staining and contact enzyme experiments. The characteristics of these strains were shown in Table 1, five of the 22 strains grew at 16 °C and one at 30 °C, most of them grew well at 20 °C, these isolates were belonged to the category of cold-adapted. The sugar fermentation metabolism was clustered by NTSYS-pc 2.01 software (Fig. 2). These isolates were classified into 5 groups (A-E) at 0.685 similarity levels, according to the results of carbohydrate utilization testing using API 50 CHL medium. Cluster A was composed of four *Lb. plantarum* all of which utilized sucrose, glucose, sorbose, raffinose and mannitol. Cluster B included five *Lb. plantarum* and two *Lb. sakei*, the strain M-CGC-1 could utilize all sugars and alcohols as carbon sources. Cluster C contained two isolates identified as *Lb. sakei* and only two strains in the D cluster were identified as *Lb. sakei* and *Lb. casei*, respectively. Cluster E was composed of four *Lb. sakei*, two *Lb. casei* and one *Lb. paracasei*. Among them, strains *Lb. sakei* (M-JYC-3, M-DGM-2) get together and the carbon source metabolism was similar, at the same time, strains *Lb. paracasei* LH-M-1 and *Lb. casei* LB-R-4-B could utilize the same carbon source but with the different strain levels.

**Table 1:** Characteristics of 22 lactobacilli with antibacterial ability isolated from the intestine of cold-water fishes

Strains	Fish species	Colony description	Growth temperature rang(°C)	Phylogenetic affiliations	
				Closest relative species	Identity (%)
CQ-CGC-2	*Leuciscus waleckii*	Rod, white, small	4–20^a^-37	*Lactobacillus plantarum*	100
M-CGC-1	*Acipenser ruthenus*	Rod, white, small	4–20-37	*Lactobacillus plantarum*	99
E-DMHM-1	*Oncorhynchus keta*	Rod, yellow, small	4–20-37	*Lactobacillus plantarum*	99
M-DMHM-3	*Oncorhynchus keta*	Rod, yellow, small	4–20-37	*Lactobacillus plantarum*	99
E-HLM-3	*Perca fluviatilis*	Rod, white, small	4–16-24	*Lactobacillus plantarum*	100
CQ-GYM-3	*Siniperca chuatsi*	Rod, white, small	4–16-24	*Lactobacillus plantarum*	100
LH-M-3	*Acipenser ruthenus*	Rod, white, medium	4–16-37	*Lactobacillus plantarum*	98
BM-M-2	*Leuciscus baicalensis*	Rod, white, small	4–20-37	*Lactobacillus plantarum*	98
LB-R-3	*Schizothorax taliensis*	Rod, white, small	4–30-37	*Lactobacillus plantar*um	98
M-BYC-1	*Erythroculter ilishaeformis*	Rod, white, small	4–20-37	*Lactobacillus sakei*	99
M-DGM-2	*Tinca tinca*	Rod, yellow, small	4–16-37	*Lactobacillus sakei*	99
M17-JYC-2	*Esox reicherti*	Rod, white, medium	4–20-37	*Lactobacillus sakei*	99
M-XLC-1	*Brachymystax lenok*	Rod, white, medium	4–20-37	*Lactobacillus sakei*	99
M-JYC-3	*Carassius auratus gibelio*	Rod, gray, small	4–20-37	*Lactobacillus sakei*	100
M-QETM-1	*Esox lucius*	Rod, white, small	4–16-37	*Lactobacillus sakei*	99
M-QETM-2	*Esox lucius*	Rod, white, small	4–20-37	*Lactobacillus sakei*	99
M17-XLC-3	*Brachymystax lenok*	Rod, white, small	4–20-37	*Lactobacillus sakei*	99
E-TZM-3	*Macropodus opercuiaris*	Rod, white, medium	4–20-37	*Lactobacillus sakei*	99
LB-R-4-B	*Schizothorax taliensis*	Rod, white, small	4–20-37	*Lactobacillus casei*	99
LC-M-11-B	*Rasbora borapetensis*	Rod, white, small	4–20-37	*Lactobacillus casei*	100
LH-R-8	*Acip*e*nser ruthenus*	Rod, white, medium	4–20-37	*Lactobacillus casei*	99
LH-M-1	*Lucioperca lucioperca*	Rod, white, small	4–20-37	*Lactobacillus paracasei*	98

^a^ indicate the optimum growth temperature

### Phylogenetic analysis of 22 representative lactobacilli

**Fig. 3:**
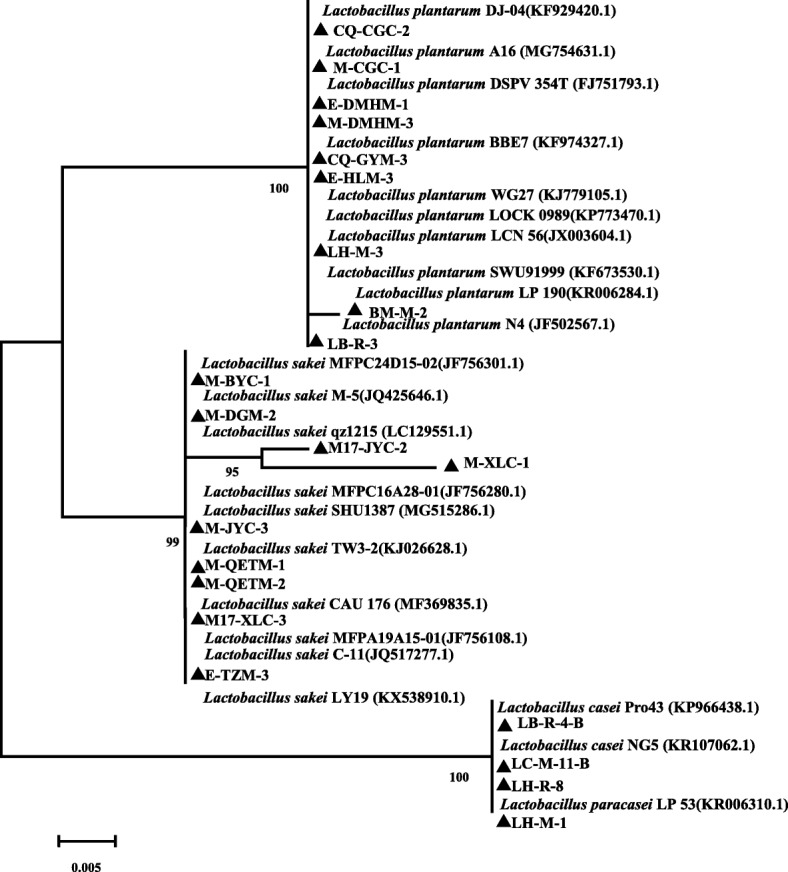
Phylogenetic tree of 22 *Lactobacillus* strains based on Neighbor-Joining distance analysis of 16S rRNA gene sequences

The phylogenetic tree based on partial 16S rRNA gene sequences for the 22 representative strains supported the clear division between these strains (Fig. 3). BLAST searches showed that these isolates recovered from various cold-water fishes exhibited very high similarity percentage values of 98~100% to their nearest described relatives deposited in GenBank, thus suggesting the close relatedness between these lactobacilli isolates and the already-described species. The first clade included nine strains closely related to *Lb. plantarum* (similarity percentage values of 100), the second clade of isolates were closely related to *Lb. sakei*. The strains (LB-R-4-B, LC-M-11-B and LH-R-8) had 100% homology with known species *Lb. casei*, another strain LH-M-1 (100% similarity) were closely related to *Lb. paracasei*, but it was clustered with *Lb. casei* in the same clade. It could be clearly seen that the 16S rRNA gene phylogenetic analysis couldn’t distinguish between *Lb.* paracasei and *Lb*. casei.

### Antimicrobial activity

Antibacterial activity against pathogenic bacterias in vitro may be considered an ideal characteristic of some probiotics. In this research, twenty-two of the 43 strains tested by the Oxford Cup method had significant antibacterial activity against six common pathogenic bacteria (Table 2). All of the strains could inhibit *Escherichia coli* EPEC O 127:K63 (CICC 10411) and *Escherichia coli* EHEC O157:H7 (CICC 21530) with the strong bacteriostatic ability, and interestingly, the strains *Lb. sakei* (M17-XLC-3, M-DGM-2, M-XLC-1), *Lb. casei* (LC-M-11-B, LB-R-4-B) and *Lb. plantarum* (E-HLM-3, BM-M-2) could inhibit all pathogenic bacteria in this experiment which had a broad spectrum of inhibition. To compare with that, *Lb. plantarum* (M-CGC-1, E-DMHM-1, CQ-GYM-3) and *Lb. sakei* (M-JYC-3, M-BYC-1) had no inhibitory effect on *Salmonella enterica subsp. enterica serovar Typhimurium* (CICC 10420) and *Salmonella enterica subsp. enterica* (CGMCC 1.10754), only the strain M-JYC-3 inhibited two common bacteria.

**Table 2:** Inhibition of *Lactobacillus* on six common pathogens isolated from the cold-water fishes intestine

Strain number	Indicative bacteria					
	CICC10411	CICC21530	CICC10420	CGMCC1.10754	CGMCC1.9136	CGMCC1.2990
M17-XLC-3	24.00 ± 0.10	19.97 ± 0.06	14.03 ± 0.15	12.00 ± 0.10	14.67 ± 4.53	11.97 ± 0.06
M-DGM-2	20.03 ± 0.06	18.00 ± 0.10	14.03 ± 0.06	14.03 ± 0.06	14.83 ± 0.25	14.03 ± 0.06
LC-M-11-B	30.03 ± 0.25	24.10 ± 0.10	20.03 ± 0.06	18.07 ± 0.12	19.50 ± 0.50	12.03 ± 0.15
E-HLM-3	24.07 ± 0.06	17.80 ± 0.26	13.03 ± 0.06	10.03 ± 0.06	15.93 ± 0.12	14.10 ± 0.36
LH-M-1	25.93 ± 0.21	21.97 ± 0.15	11.93 ± 0.31	–	14.80 ± 0.53	12.20 ± 0.72
M-BYC-1	24.07 ± 0.12	24.07 ± 0.12	–	–	15.87 ± 0.42	13.97 ± 0.06
M-CGC-1	23.10 ± 0.10	19.57 ± 0.45	–	–	20.33 ± 1.53	14.03 ± 0.15
LB-R-3	24.17 ± 0.29	23.17 ± 0.21	13.87 ± 0.32	–	11.83 ± 0.29	13.17 ± 0.29
LB-R-4-B	25.13 ± 0.15	22.97 ± 0.15	22.03 ± 0.06	14.10 ± 0.10	14.00 ± 0.20	14.00 ± 0.20
BM-M-2	22.83 ± 0.15	26.13 ± 0.23	15.03 ± 0.15	15.90 ± 0.10	13.00 ± 0.10	12.10 ± 0.17
M17-JYC-2	22.00 ± 0.15	19.93 ± 0.21	18.03 ± 0.25	11.97 ± 0.15	–	13.80 ± 0.17
LH-M-3	23.00 ± 0.10	27.10 ± 0.10	13.13 ± 0.23	10.07 ± 0.12	11.67 ± 0.58	12.03 ± 0.06
M-QETM-1	25.00 ± 0.10	24.03 ± 0.06	16.00 ± 0.10	14.27 ± 0.25	12.80 ± 0.20	19.53 ± 0.50
M-QETM-2	23.90 ± 0.10	26.07 ± 0.21	12.93 ± 0.12	–	14.97 ± 0.25	12.93 ± 0.12
M-DMHM-3	22.07 ± 0.12	21.10 ± 0.10	14.13 ± 0.15	–	11.87 ± 0.23	11.87 ± 0.23
E-DMHM-1	25.10 ± 0.10	18.93 ± 0.21	–	–	14.83 ± 0.29	14.20 ± 0.26
M-XLC-1	27.00 ± 0.20	22.10 ± 0.17	15.10 ± 0.10	14.03 ± 0.06	15.10 ± 0.10	15.10 ± 0.10
E-TZM-3	22.13 ± 0.15	20.07 ± 0.06	–	13.10 ± 0.17	13.83 ± 0.15	13.83 ± 0.15
CQ-CGC-2	25.03 ± 0.06	22.07 ± 0.12	13.07 ± 0.12	–	12.00 ± 0.20	9.80 ± 0.53
LH-R-8	24.10 ± 0.17	29.97 ± 0.06	13.93 ± 0.21	17.17 ± 0.29	–	9.83 ± 0.29
M-JYC-3	23.90 ± 0.10	25.03 ± 0.25	–	–	–	–
CQ-GYM-3	22.00 ± 0.06	19.97 ± 0.15	–	–	12.90 ± 0.26	17.50 ± 0.50

“−“indicates no suppression; The values in the table are the diameter of the zone of inhibition (unit: mm), and the values are the mean; ± standard deviation of the three tests

### Results of tolerance test of lactic acid bacteria

**Fig. 4:**
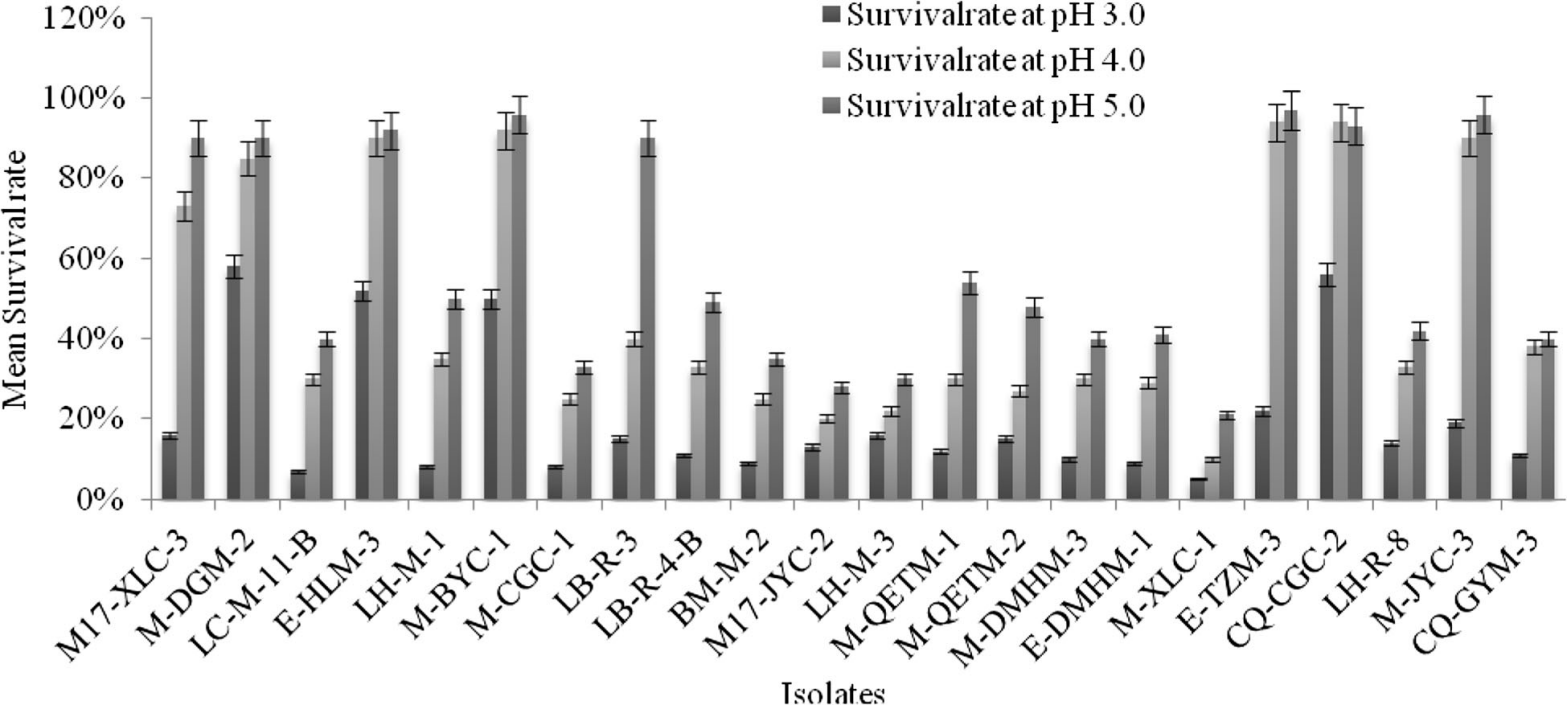
A survival rates of lactobacilli strains after incubation at pH values 3.0, 4.0, and 5.0

**Fig. 5:**
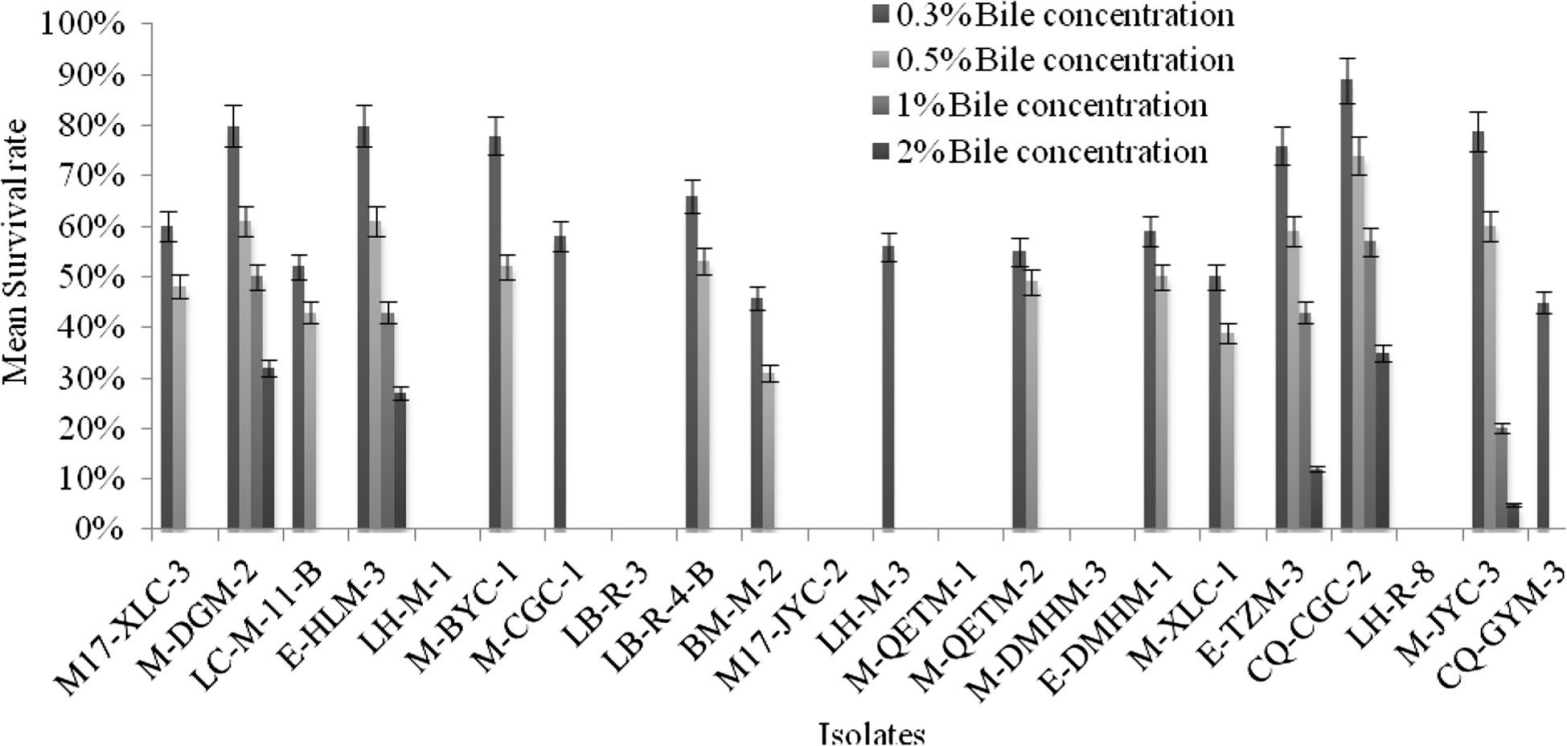
Survival rate of lactobacilli strains incubation in different bile salt concentrations (0.3, 0.5, 1, and 2%)

As shown in the Figs. 4 and 5, the test strains were grown in modified MRS medium with different pH values and bile salt concentrations, the number of colonies on the plate was used to determine the survival rate. Eight of the twenty-two strains had a survival rate of more than 90% at pH 5.0 and seven strains showed above 70% of the survival rate at pH 4.0. Only four of the strains showed more than 50% of survival rate at pH 3.0. i.e. *Lb. sakei* strains (M-DGM-2, M-BYC-1) and *Lb. plantarum* (E-HLM-3, CQ-CGC-2) have shown maximum survivability at low pH as a comparison to the other strains. In the meantime, the results showed that the survival rate decreases with a rise in bile salt concentrations. It can be seen from the growth condition that sixteen strains were able to survive at a bile salt concentration of 0.3%, with the gradual increase of bile salt concentrations, the growth of most lactobacilli were inhibited, but *Lb. sakei* M-DGM-2 and *Lb. plantarum* (E-HLM-3, CQ-CGC-2) demonstrated the maximum survival rate, they tolerated even 2% bile concentration.

### Susceptibility to antibiotics

**Fig. 6:**
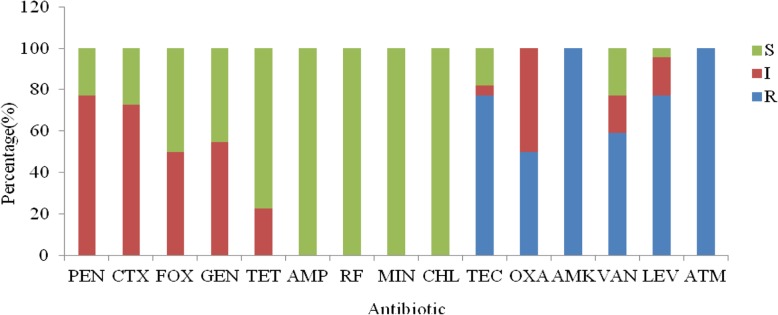
Antibiotic resistance of 22 strains of lactobacilli with antibacterial activity. PEN: penicillin; AMP: ampicillin; OXA: oxacillin; CTX: cefotaxime; FOX: cefoxitin; GEN: gentamicin; AMK: amikacin; TET: tetracycline; MIN: minocyline; CHL: chloramphenicol; RF: rifampicin; VAN:vancomycin; TEC: teicoplanin; LEV: levofloxacin; ATM: aztreonam. The total number of investigated *Lactobacillus* strains was taken as 100%. S: sensitive, I: intermediately resistant, R: resistant

For the hemolysis, none of the *Lactobacillus* strains was able to hydrolyze sheep blood, proving non hemolytic activity. The resistance or sensitivity of the 22 *Lactobacillus* with antibacterial ability strains isolates to the selected antibiotics is shown in Fig. 6. The results of the drug sensitivity test were mainly measured in accordance with the CLSI 2014 standard [16]. The sensitivity of lactobacilli to antibiotics of different classes was evaluated: penicillins (penicillin, ampicillin, and oxacillin), aminoglycosides (amikacin, gentamycin), cephalosporin (cefotaxime, cefoxitin), telracyclies (tetracycline, minocyline), glycopeptides (vancomycin, teicoplanin), aztreonam, chloramphenicol, levofloxacin, and rifampicin. The studied lactobacilli showed sensitivity to telracyclies (tetracycline, minocyline), chloramphenicol, rifampicin, ampicillin and were resistant to glycopeptides (vancomycin, teicoplanin), levofloxacin, aztreonam, amikacin and oxacillin. At the same time, the *Lactobacillus* strains showed semi-tolerant to other studied antibiotics.

**Fig. 7:**
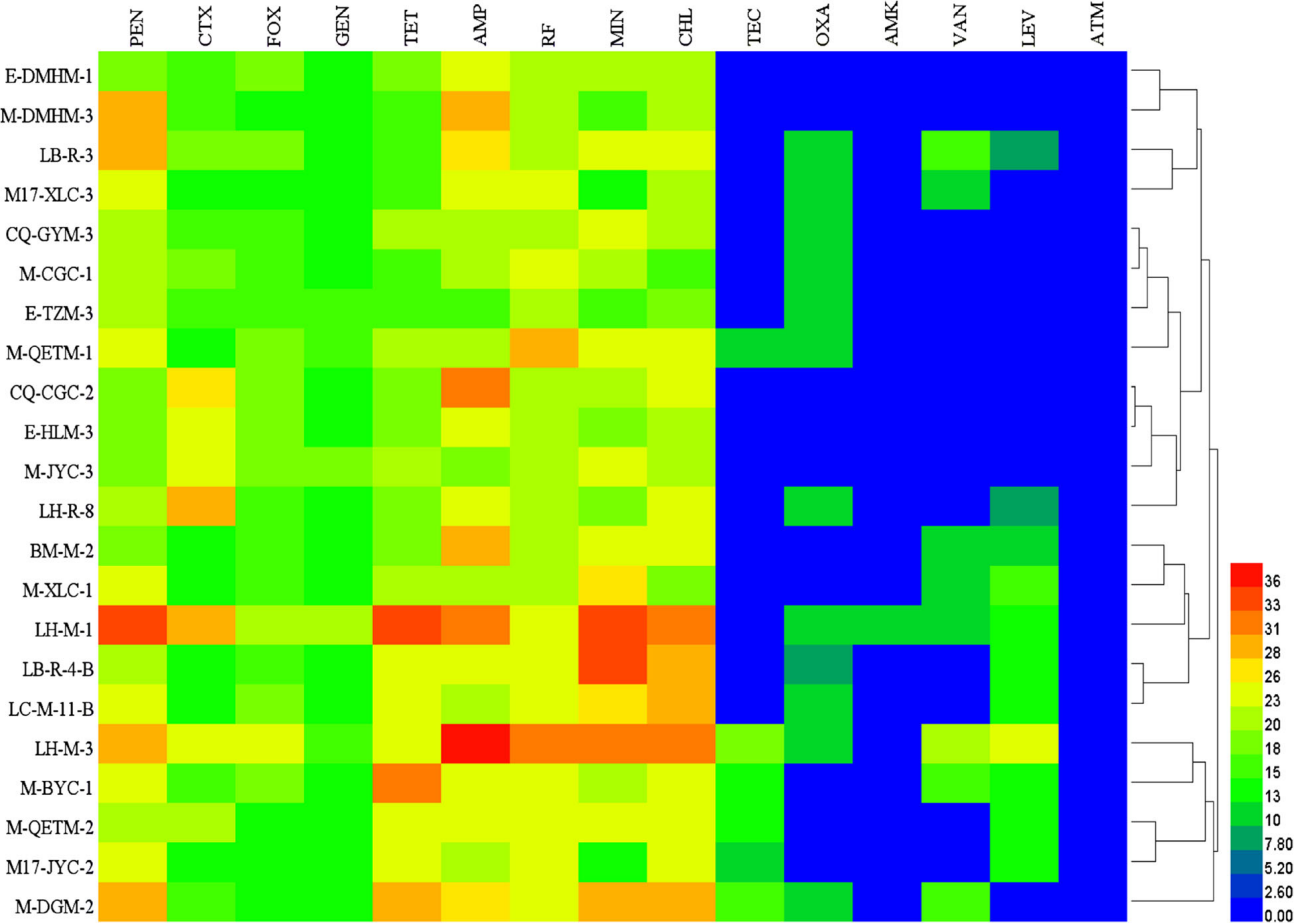
Heat map analysis of antibiotic resistance of 22 strains of lactobacilli with bacteriostatic properties based on diameter of inhibition zone. PEN: penicillin; AMP: ampicillin; OXA: oxacillin; CTX: cefotaxime; FOX: cefoxitin; GEN: gentamicin; AMK: amikacin; TET: tetracycline; MIN: minocyline; CHL: chloramphenicol; RF: rifampicin; VAN:vancomycin; TEC: teicoplanin; LEV: levofloxacin; ATM: aztreonam. All sorts of color in figure represents the size of the bacteriostatic circle diameter, red indicates the largest diameter of the inhibition zone, blue represents the bacteriostatic circle diameter of the minimum, from blue to red in the process of gradient increase the diameter of bacteriostatic ring in turn

Heat map Fig. 7 could visually reflect the diameter of antibiotic inhibition zone produced by studied lactobacilli by color gradient, and respond to antibiotic resistance of *Lactobacillus* strains. The isolates showed different responses to the antibiotics, and most of them were sensitive or semi-resistant to 9–13 different studied antibiotics. Especially the isolates *Lb. paracasei* LH-M-1 and *Lb. plantarum* LH-M-3 showed sensitive to or semi-tolerance of 13 (7 + 6) and 13 (10 + 3) different antibiotics, respectively, were the most sensitive isolates. All the studied isolates were resistant to 2–6 different studied antibiotics. It is worth noting that lactobacillus as probiotics have different drug resistance to similar antibiotic drugs, such as gentamicin and amikacin in aminoglycosides, and benzocillin in penicillin antibiotics are significantly different from those in penicillin and ampicillin. Previous research has shown that the resistance of lactic acid bacteria to aminoglycosides is considered to be natural and associated with a low permeability of lactobacilli cells to antibiotics of this group. Members of the genus *Lactobacillus* are usually resistant to glycopeptides potentiates the risk of spread of antibiotic resistance genes in the environment because genes of vancomycin and/or teicoplanin resistance are often located on mobile genetic elements, including conjugative plasmids and transposons.

## Discussion

Conventional phenotypic and physiological tests have been used on different intestinal microbial isolates to analyze and rapidly identify microbial communities [17]. Although these conventional methods have proven to be useful and indispensable tools for characterizing LAB, they are limited in identification ability and accuracy [18, 19]. Therefore, a combination of phenotypic and molecular biological methods has become the preferred way to determine and analyze the species composition of target microbial communities [20]. Several studies have reported success using random amplification of polymorphic DNA-PCR to differentiate LAB strains [21–23]. In this regard, PCR-based genomic fingerprinting technology is currently developing rapidly and is widely used in a variety of bacterial gene classifications, as it is easy to implement [24, 25]. Effective and accurate methods for identifying and discriminating cold-water fish intestinal lactobacilli are needed, to quickly identify species and promote the study of complex flora in the intestinal ecosystem. Characterization of 43 selected lactobacilli isolates was initially conducted by cell morphology, gram staining, contact enzyme experiments, and carbohydrate fermentation tests. Analyses using API 50 CHL medium are not entirely consistent with the phenotypic identification of fish gut lactobacilli, and the present results revealed the limits of this approach. However, the combined results of traditional phenotypic tests, the Rep-PCR identification technique and 16S rRNA gene sequencing of representative strains showed that the majority of isolates from cold-water fishes consisted of *Lb. plantarum*, *Lb. sakei*, *Lb. casei*, and *Lb. paracasei*, and the optimum growth temperature of these strains was 16–20 °C, which were initially considered to be cold-adapted strains [26, 27]. The Rep-PCR technology amplifies the entire bacterial genome, so this method accurately reflects the genetic diversity of a bacterial population, with high resolution, and can distinguish LAB at the species, subspecies, and strain levels. Therefore, this study used a Rep-PCR fingerprint cluster analysis with the BOXA 1R and (GTG)_5_ primers to identify *Lactobacillus* spp. The results of our study show that the fingerprint patterns produced by PCR using the BOXAIR and (GTG)_5_ primers were rich, and the isolates were well distinguished, similar to those of Urraza et al. [28]. The two *Lactobacillus* species were closely related taxonomically and difficult to differentiate by a BLAST search against GenBank, whereas the 16S rRNA gene was occasionally limited to species-level identification, including *Lb. casei* and *Lb. paracasei* [29]. Interestingly, the Rep-PCR fingerprinting technology could well distinguish between *Lb. casei* and *Lb. paracasei.*

It is important to select lactobacilli strains with probiotic potential to balance the microorganisms in the GI tract and provide additional health benefits. *Lactobacillus*, which is an important probiotic group in the guts of humans and animals, is a gram-positive, non-sporogenic, anaerobic, or facultative anaerobic bacteria that produces large amounts of organic acids during growth, while some strains also produce specific lactobacillin, which have biological antiseptic properties, and may prevent and inhibit the invasion and colonization of pathogenic microorganisms by biological antagonism or by reducing pH [30, 31]. This experimental study detected 22 representative lactobacilli strains selected according to the bacteriostatic test with common pathogens, such as *Escherichia coli* EPEC O 127:K63 (CICC 10411), *Escherichia coli* EHEC O157:H7 (CICC 21530), *Listeria monocytogenes* (CGMCC 1.9136) and *Listeria innocua* (CGMCC 1.2990), which have the strongest bacteriostatic effect, while the bacteriostatic action against *Salmonella enterica subsp. enterica serovar Typhimurium* (CICC 10420), and *Salmonella enterica subsp. enterica* (CGMCC 1.10754) was relatively weak. In short, the 22 lactobacillus strains detected in this study have the ability to inhibit the growth of pathogenic bacteria. The ability of lactobacilli to inhibit such pathogenic bacteria is due to the nature of the organic acids they produce as well as other antimicrobial compounds, such as hydrogen peroxide, bacteriocins, and diacetyl, which enhance the inhibitory activity of lactic acid [32, 33]. Determining which substances specifically inhibit the growth of pathogenic bacteria requires further study to lay the foundation for use of *Lactobacillus* to treat of GI diseases in humans and animals [34, 35]. Tolerance to low pH and bile- salts are the most important criteria when accepting a microorganism as a probiotic. Among the 22 strains of *Lactobacillus* spp. with bacteriostatic properties in the present study, maximum survival rate (> 50% viability) was achieved by only four strains at pH 3.0 after plate counting, suggesting that lowering the pH below 5.0 reduces the viability of lactobacill strains. Our results corroborate the results obtained by Wang et al. [36]. In addition to pH, bile salt tolerance has been considered a condition for selecting lactobacillus strains. The majority of the lactobacillus strains survived (55–80% viability) a 0.3% bile salt concentration, whereas only four isolates survived at a concentration of 2% bile salts, and all other strains were resistant to bile. These observations were consistent with Fang et al. [37].

One of the most important properties of probiotic bacteria is a safety assessment of antibiotics. China is a large aquaculture country, and intensive fish culture provides a good opportunity for rapid propagation of pathogenic bacteria [38]. The incidence of bacterial diseases in aquaculture is increasing annually and a large number of antibiotics are used to prevent and control these diseases. However, the widespread use of antibiotics can lead to antibiotic resistance by some pathogens [39]. Broughton et al. isolated strains from 100 freshwater fish in Guangdong and found that all strains were resistant to erythromycin and ampicillin [40]. Hoque et al. [32, 41] reported that *Lactobacillus* strains are sensitive to amoxicillin, gentamycin, clindamycin, and azithromycin and resistant to kanamycin, cefradine, and tetracycline. The present study evaluated the resistance of 15 antibiotics to lactobacilli isolated from the intestinal tract of wild cold-water fish in Xinjiang. The studied lactobacilli showed sensitivity to or semi-tolerant to telracyclies (tetracycline, minocyline), chloramphenicol, rifampicin, ampicillin or penicillin, cefotaxime, cefoxitin, gentamicin, and tetracycline, while all strains were resistant to glycopeptides (vancomycin, teicoplanin), levofloxacin, aztreonam, amikacin and oxacillin. The sensitivity of the *Lactobacillus* strains in this study was likely related to the water layer and benthic life of cold-water fishes. Drug resistance can be related to fish habit, food chain grade, and product characteristics, but the specific reasons need further study [42]. The safety of *Lactobacillus* strains in the cold-water fishes was moderate, but some strains may have their own natural resistance to antibiotics. In future study, we will further investigate the probiotic characteristics and the related resistance genes of cryogenic lactobacilli, to provide a scientific basis for applying of cold-adapted lactobacilli as probiotics in fish feed additives, and lay a theoretical foundation for fresh food technology.

## Conclusion

Our findings revealed that 43 *Lactobacillus* strains isolated from the intestinal tract of cold-water fishes in Altay, Xinjiang had abundant genetic diversity, and there were excellent genetic polymorphisms in intraspecific differences. According to the bacteriostatic test, twenty-two *Lactobacillus* strains (growth at optimal temperature range of 16~20 °C) showed a wide range of antimicrobial activity against 6 different pathogenic strains, and none of the strains tested appeared to be haemolytic. There were three lactobacilli isolates E-HLM-3, CQ-CGC-2, M-DGM-2 might be used as probiotic bacteria (these isolates showed quite high antimicrobial activity, and antibiotic resistance, at the same time with the good acid and bile salt resistance). Thus, these strains were good candidates to be used as new food preservative agents and/or probiotic additives in feeding of aquaculture.

## Materials and methods

### Sample collections and bacterial isolates

Eerqisi River is located in the Altay region of northern part of Xinjiang, China (45°00′~ 49°10′ N, 85°31′~ 91°01′ E), which is geographically classified as cold-temperate zone. There are more than 10 indigenous wild cold-water fishes in the lakes and rivers where the average annual temperature ranges from 5 to 8 °C, and the water temperature is less than 20 °C all the year round. Fish samples (sixteen kinds of healthy, adult cold-water fishes, their species names are shown in Table 1) were carried out in the month of August of two consecutive years (2014 and 2015). Which were procured from a local cold-water fishes breeding bases of Altay, Xinjiang, and transported in ice-packed boxes to the Biotechnology Laboratory, Food College, Shihezi University. This study did not involve any protected or endangered species, hence no specific permissions and ethics were required to collect the fish samples. However, all animal studies were performed in accordance with ARRIVE guidelines, with the approval of the Ethics of Animal Experiments of Shihezi University Committee, Shi Hezi, China. The pathogenic bacterial strains *Escherichia coli* (EPEC) O127:K63 (CICC 10411), *Escherichia coli* (EHEC) O157:H7 (CICC 21530), *Listeria monocytogenes* (CGMCC 1.9136), *Listeria innocua* (CGMCC 1.2990), *Salmonella enterica subsp. enterica serovar Typhimurium* (CICC 10420) and *Salmonella enterica subsp. enterica* (CGMCC 1.10754) used in the study for antibacterial activity were purchased from China Industrial Microbial Species Conservation Management Center (Wuhan, China).

One gram of the intestinal samples of cold-water fishes were diluted by 10 times gradient in turn, and the bacterial suspensions of 10^− 2^, 10^− 3^ and 10^− 4^ gradients were uniformly coated on MRS medium and cultured at 16 °C for 2–5 days under microaerophilic conditions. Single bacterial colonies on the surface of the plate were selected and separated by streaking, and the cells were cultured for 3 to 4 times until purification. Gram staining and contact enzyme experiments were carried out to screen suspected lactic acid bacteria. All the purified strains were centrifuged then suspended in fresh medium, and supplemented with 50% (w/v) sterile glycerol, and stored frozen in a − 80 °C freezer for further analysis.

### Phenotypic characterization

Cell morphology of all isolates and their motility were determined using a phase contrast microscope (Olympus CX21, Japan). Overnight cultures of isolates grown on MRS agar were submitted to gram staining and tested for catalase production as described by Okada et al. [43]. All the isolates matched to the basic traits of the LAB group, non-spore forming, gram-positive, and catalase negative were considered for identification. Sugar fermentation patterns of partial LAB isolates were determined using the API 50 CHL (bioMérieux, France) medium for culturing lactobacilli. In the system analysis, the carbon source metabolism was transformed into a matrix containing only 1 and 0 two-valued variables, and cluster analysis was performed using NTSYS-pc 2.01 (Applied Biostatistics Inc., New York, USA) software, using a non-weighted arithmetic average linkage method. The optimum growth temperature of the *Lactobacillus* strains were determined by measuring the OD at 420 nm after incubation for 24 h at 4 °C, 10 °C, 16 °C, 20 °C, 25 °C, 30 °C and 37 °C. The experiment was repeated three times and a blank control was performed.

### Rep-PCR genomic fingerprinting

DNA from the *Lactobacillus* spp. was extracted according to the urea-SDS-NaOH method [44] with slight modifications.

Rep-PCR was performed according to the methods of Lee et al. [45]. The fingerprints were analyzed by single primer BOXA 1R and (GTG)_5_, and the PCR reaction conditions were shown in Table 3. PCR products were separated by electrophoresis on a 2.0% (w/v) agarose gels using 1 × TAE buffer (2 mol/L Tris base, 1 mol/L acetic acid, 0.05 mol/L EDTA with pH 8.0) and run at 80 V for 3.5 h. The gels were photographed on a UV transilluminator (Power Pas Universal, BioRad, USA) and the resulting images were saved in TIFF format. The resulting Rep-PCR fingerprints were analyzed using the Gel Compar II 6.0 software (Applied Maths, Austin, TX, USA). Pearson coefficients were used for similarity analysis and UPGMA output was used for dendrogram.

**Table 3:** Primers and PCR conditions used in rep-PCR

Method	Primer	Sequence (5′-3′)	PCR conditions		
			Denaturation	Annealing	Extension
BOX-PCR	BOXA 1R	CTACGGCAAGGCGACGCTGACG	94 °C, 1 min	53 °C, 1 min	65 °C, 8 min
(GTG)_5_-PCR	(GTG)_5_	GTGGTGGTGGTGGTG	94 °C, 30 s	50 °C, 30 s	65 °C, 8 min

### 16S rRNA amplification and sequencing

The 16S rRNA gene was amplified using gene universal primers 27 forward primer (FP) (5′-AGAGTTTGATCCTGGCTCAG-3′) and 1492 reverse primer (RP) (5′-TACGGCTACCTTGTTACGACTT-3′). The amplification of the gene was performed in a 25 μL reaction mixture for PCR containing 3 μL of the DNA template (approximately 20 ng/μL), 2 × Taq MasterMix 12.5 μL, 0.5 μL (0.4 μM/mL) of each primer, and with ddH_2_O supplemented to 25 μL. The PCR conditions consisted of 35 cycles (pre-denaturation at 95 °C for 5 min; denaturation at 95 °C for 1 min, annealing at 53 °C for 1 min, and elongation step at 72 °C for 1 min), and one additional cycle at 72 °C for 7 min. The PCR was carried out in a thermocycler PCR System (TC-512, Techne, U.K.) and the 16S rRNA amplicons were analyzed by electrophoresis on 1.5% (w/v) agarose gels with 5 mL of Gel Stain (Invitrogen, Life technologies, USA), followed by 100 V for 60 min in 1 × TAE buffer and visualized by UV light. Observed and photographed the bands under the gel imaging system, the PCR products were sent to Shanghai Shenggong Company for sequencing. The 16S rRNA gene sequences closer to the genetic relationship from the GenBank database was aligned using CLUSTAL X 1.83 software. The evolution distance was calculated using the neighbor-joining method. In the MEGA version7.0 [46], the evolution tree was constructed using the p-distances and the Kimura-2 parameter double-parameter method. The stability of the branching pattern of the evolution tree was bootstrap method, and the number of repetition was 1000.

### Antibacterial assay

The *Lactobacillus* strains were added to the modified MRS liquid medium at 1% inoculation amount, cultured at 20 °C for 24 h, centrifuged at 10, 000 r/min for 5 min, and then the supernatant was filtered through a 0.22 μm filter (Millipore Ltd., Watford, UK) to obtain cell-free supernatants (CFS). *Escherichia coli* EPEC O 127:K63 (CICC 10411), *Escherichia coli* EHEC O157:H7 (CICC 21530), *Salmonella enterica subsp. enterica serovar Typhimurium* (CICC 10420), *Salmonella enterica subsp. enterica* (CGMCC 1.10754), *Listeria monocytogenes* (CGMCC 1.9136), *Listeria innocua* (CGMCC 1.2990) were used as indicator bacteria for bacteriostatic experiments. The antimicrobial activities of CFS produced from isolated bacteria were tested using the Oxford cup method [47]. These indicator bacteria were inoculated in LB, PYG and TSA medium (Hai Bo Biotechnology, Qingdao, China), respectively. After 18 h of incubation at 37 °C, the suspension with 100 mL (concentration of about 10^6^–10^7^ cfu/mL) was evenly coated on the surface of severally medium with sterile cotton swabs. The oxford cup was gently pressed on the coated medium, and added 200 μL of cell-free fermentation supernatant to the cup, using non-bacterial MRS medium as blank control, then pre-diffused 5 h at 4 °C, and incubated at 37 °C for 18 h to determine the diameter of the inhibition zone. Three parallel experiments were conducted in each experiment to determine the bacteriostasis ability.

### The pH and bile tolerance

Acid resistance of LAB strains were assayed using the method of Owusu-Kwarteng et al. [48] with modification. The strains were grown overnight (24 h) on MRS broth at 24 °C, the cultured cell fluid was centrifuged at 5000×g for 5 min at 4 °C and washed with phosphate-buffered saline (PBS) solution (0.1 M, pH 7.2) in twice. The cell pellet was re-suspended in PBS solution with different pH, i.e., 3.0, 4.0, and 5.0 incubate for 4 h. Then absorbed 100 μL of these dilutions were applied to MRS agar plates at 24 °C for 24 h, and the number of colonies on the plate was used to determine the survival rate (the viable cell count was recorded and compared with initial viable cell count). For bile salt tolerance, survival rates for these strains were estimated at different bile salt concentrations (0.3, 0.5, 1, and 2%). The experiment was carried out using the same experimental method as the acid resistance test and compared with the control. In this case, the experiment was conducted in triplicates and averaged.

### Haemolytic activity

Haemolytic activity was determined by streaking the *Lactobacillus* strains on the blood agar plate, containing 5% sheep and incubated for 48 h at 37 °C. The haemolytic reactions were examined for signs of β-haemolysis (clear halo around colonies), α-haemolysis (green halo around colonies) or γ-haemolysis (no halo around colonies).

### Susceptibility to antibiotics

The *Lactobacillus* strains with antibacterial activity were tested for resistance to antibiotics by the disc diffusion method, according to Fortina et al. [49, 50]. The prepared 1 × 10^8^ cfu/g suspension was picked up with a sterile cotton swab and evenly spread on the surface of the MRS medium, and then the drug-responsive papers were tightly fixed on the surface of the medium with sterile tweezers. Microaerophilic culture was performed at 20 °C for 18–24 h to determine the diameter of the inhibition zone with a digital calliper (Absolute Digimatic Caliper, Mitutoyo, USA). Antibiotic discs (Oxoid, England) were used to determine the susceptibility of the strains to 15 antibiotics: minocyline (30 μg/disc), chloramphenicol (30 μg/disc), amikacin (30 μg/disc), ampicillin (10 μg/disc), gentamicin (120 μg/disc), teicoplanin (30 μg/disc), cefotaxime (30 μg/disc), cefoxitin (30 μg/disc), oxacillin (1 μg/disc), levofloxacin (5 μg/disc), aztreonam (30 μg/disc), penicillin (10 μg/disc), rifampicin (5 μg/disc), tetracycline (30 μg/disc), and vancomycin (30 μg/disc), analyses were done in duplicate. Each isolate was classified as resistant (R), sensitive (S) and/or intermediate (I) according to the inhibition zone diameters in agreement with the Clinical and Laboratory Standards Institute tables [51].

### Statistical analyses

Values from each trial were determined from means of duplicate data, the consequence of sugar fermentation metabolism of *Lactobacillus* spp. were clustered by NTSYS-pc 2.01 software and the Rep-PCR fingerprints of the strains were analyzed using Gel Compar II 6.0 software. The collected data from tolerance to pH and bile salts were performed with Microsoft Excel 2010. Meanwhile the average and standard deviation of the results of bacterial inhibition were calculated to represent the inhibition zone diameter for each *Lactobacillus* strain.

## Data Availability

All data and materials are available on request for academic use.

## Abbreviations

AMK: Amikacin
AMP: Ampicillin
ATM: Aztreonam
CHL: Chloramphenicol
CTX: Cefotaxime
FOX: Cefoxitin
GEN: Gentamicin
I: Intermediately resistant
*Lb.*: *Lactobacillus.*
LEV: Levofloxacin
MIN: Minocyline
OXA: Oxacillin
PEN: Penicillin
R: Resistant
Rep-PCR: Repetitive extragenic palindromic polymerase chain reaction
RF: Rifampicin
S: Sensitive
TEC: Teicoplanin
TET: Tetracycline
VAN: Vancomycin
